# Hair analysis following chronic smoked-drugs-of-abuse exposure in adults and their toddler: a case report

**DOI:** 10.1186/1752-1947-5-570

**Published:** 2011-12-10

**Authors:** Esther Papaseit, Xavier Joya, Marta Velasco, Ester Civit, Pau Mota, Marta Bertran, Oriol Vall, Oscar Garcia-Algar

**Affiliations:** 1 Programa de Recerca en Neurociències, IMIM (Institut de Recerca, Hospital del Mar), Parc de Salut Mar, Barcelona, Spain; 2 Grup de Recerca i Entorn (GRIE), Institut de Recerca, Hospital del Mar, Parc de Salut Mar, Barcelona, Spain; 3 Universitat Autónoma de Barcelona, Barcelona, Spain

## Abstract

**Introduction:**

Over the past two decades, the study of chronic cocaine and crack cocaine exposure in the pediatric population has been focused on the potential adverse effects, especially in the prenatal period and early childhood. Non-invasive biological matrices have become an essential tool for the assessment of a long-term history of drug of abuse exposure.

**Case report:**

We analyze the significance of different biomarker values in hair after chronic crack exposure in a two-year-old Caucasian girl and her parents, who are self-reported crack smokers. The level of benzoylecgonine, the principal metabolite of cocaine, was determined in segmented hair samples (0 cm to 3 cm from the scalp, and > 3 cm from the scalp) following washing to exclude external contamination. Benzoylecgonine was detectable in high concentrations in the child's hair, at 1.9 ng/mg and 7.04 ng/mg, respectively. Benzoylecgonine was also present in the maternal and paternal hair samples at 7.88 ng/mg and 6.39 ng/mg, and 13.06 ng/mg and 12.97 ng/mg, respectively.

**Conclusion:**

Based on the data from this case and from previously published poisoning cases, as well as on the experience of our research group, we conclude that, using similar matrices for the study of chronic drug exposure, children present with a higher cocaine concentration in hair and they experience more serious deleterious acute effects, probably due to a different and slower cocaine metabolism. Consequently, children must be not exposed to secondhand crack smoke under any circumstance.

## Introduction

Since the mid-1990s, cocaine has been the second most popular illegal recreational drug in Europe among young adults and adolescents, the first being cannabis [1]. Similar to other illicit drugs, cocaine is commonly used by different routes of administration: orally, by smoking or chewing; parenterally; as a suppository; and, mainly, by snorting or inhalation of crack cocaine smoke. Free-base crack cocaine is a potent form of cocaine rapidly absorbed through the pulmonary circulation and reaching the central nervous system within seconds, which results in rapid and striking stimulant effects [2]. Its pharmacological action by smoking is responsible for many of the reported neurological and pulmonary toxic effects [3,4]. Over the last 20 years, parallel to the advances in laboratory techniques for detecting recent drug use, hair analysis has emerged as the most important non-invasive biomarker used to provide long-term information on an individual's drug use [5-8]. Decontamination of hair samples by washing before analysis, detection of the relevant metabolites and use of adequate cut-off levels have been the main considerations in testing for drugs in hair [9]. The differentiation between systemic exposure and external contamination has been a controversial topic and one of the limitations of cocaine testing in hair [10-12]. In fact, hair exposed to crack cocaine by external contamination, where the individual does not actively consume the drug but traces may be deposited on the hair, must be carefully considered [13]. At present, hair analysis is used to monitor for drugs of abuse and environmental exposure to drugs of abuse [14]. In the pediatric population, hair screening has been used to determine gestational cocaine exposure [15,16], detect unsuspected exposure to cocaine in children [17,18] and confirm chronic cocaine exposure in cases of acute intoxication or high suspicion [19]. Similarly, in adults, cocaine hair screening is routinely applied to detect drug abuse in forensic cases, occupational and traffic medicine and clinical toxicology [14], including monitoring of cocaine-abusing households by child welfare agencies in child custody cases.

To the best of our knowledge, there are no previous publications reporting on biomarker values in children's hair after chronic exposure to crack smoke and active abuse and exposure in adults. Here, we present a case of chronic exposure to cocaine through passive inhalation of crack cocaine smoke in a two-year-old girl as result of suspicion of environmental exposure to this drug of abuse by a pediatrician.

## Case presentation

A two-year-old Caucasian girl was brought to our Pediatric Emergency Department, referred from a pediatric outpatient center for unclear reasons after presenting with respiratory symptoms for two weeks. Her medical records showed that she had been diagnosed with neonatal withdrawal syndrome, which required long term treatment with phenobarbital. At the outpatient center, she had been diagnosed with repeated bronchiolitis and she needed health care services from a pediatric pulmonologist, who prescribed treatment with fluticasone. She had no other relevant medical or pathological background.

Medical records informed us that the child's mother had a medical history of substance-related disorders and she was receiving treatment for heroin dependence with methadone. No other pharmacological treatment was prescribed for cannabis and tobacco abuse.

When attending our department, the mother presented an unhealthy aspect and abnormal behavior. A physical examination of the child showed a minor asthma attack with respiratory difficulties and clinical features refractory to the outpatient bronchodilator treatment (salbutamol) over the past 15 days. The examination also revealed use of her accessory muscles for respiration, wheezing and bilateral hypoventilation. Her blood oxygen saturation level was 98% and cyanosis was not observed. Her vital signs revealed tachycardia and tachypnea without fever (her heart rate was 148 beats per minute, her respiratory rate, 60 breaths per minute and temperature 36°C). A neurological examination did not show a focal neurological deficit (15/15 Glasgow Score Scale) and the remainder of the physical examination was normal. The child received respiratory support and pharmacological treatment with gradual clinical improvement. Her blood cell count and serum biochemistry were normal. Under suspicion of exposure to drugs of abuse, urinary toxicology screening for commonly used drugs of abuse by cloned enzyme donor immunoassay (CEDIA, Microgenics, Barcelona, Spain) was done. Repeated urinary tests were positive for cocaine. Since urine toxicology was done in a pediatric emergency ward and speed was of paramount importance, the level of benzoylecgonine (BZG) in her urine was not quantified.

Information concerning the environment where the child had been living was obtained from the mother, who confirmed a history of crack smoking by both parents in the presence of the child during the days previous to her hospital admission. She denied that her child had ingested cocaine by any other route than passive inhalation. Chronic exposure to cocaine in an environment where drugs of abuse were being used by the parents was verified by hair analysis for the child and her parents (Table 1).

**Table 1:** Hair segment concentration of drugs of abuse from the child and her parents using VMA/DRI^®^.

Hair segments	Drugs of abuse and metabolites in hair (cut off values)		
	
	BZG (0.50 ng/mg)	Cannabis (0.10 ng/mg)	Opiates (0.50 ng/mg)
Child's hair			
0 cm to 3 cm	1.9	0.3	ND
> 3 cm	7.04	0.7	ND
Mother's hair			
0 cm to 3 cm	7.88	0.92	13.88
> 3 cm	6.39	0.94	3.15
Father's hair			
0 cm to 3 cm	13.06	0.28	ND
> 3 cm	12.97	0.86	ND

BZG: benzoylecgonine; ND: none detected

After 48 hours, our patient recovered completely without any respiratory difficulties. She was discharged from the hospital and the proper authorities were alerted in order to evaluate the child's home conditions.

In the present case, a quantitative analysis of cocaine, opiates, amphetamines, cannabis and 3,4-methylenedioxymethamphetamine (MDMA) in her hair was performed according to forensic standards using an immunoassay (VMA/DRI^®^) on the available length of hair. The child's and her parent's hair samples (as an entire strand) were cut close to the scalp at the vertex region using stainless steel scissors. Whenever possible (hair shaft length more than 3 cm), the hair strands were divided into subsequent 3 cm segments, each representing hair growth in subsequent three-month periods. To exclude external contamination, the samples were washed before testing.

A comprehensive toxicological screening was performed on the collected hair samples. The test was positive for BZG, the main metabolite of cocaine, and cannabis in the entire hair samples. Quantitative analysis confirmed that BZG was present in the child's hair at a concentration of 1.9 ng/mg and 7.04 ng/mg. BZG concentrations found in her mother's and father's hair were 7.88 ng/mg and 6.39 ng/mg, and 13.06 ng/mg and 12.97 ng/mg, respectively. Opiates were detected in her mother's hair. Amphetamines and MDMA were not detected.

## Discussion

The information regarding passive postnatal toxic exposure to cocaine in children is limited. At our hospital, hair analysis in preschool children without signs or symptoms suggestive of exposure revealed an estimated prevalence of exposure of 23.3% [17]. There are several clinical case reports detailing concentrations of BZG in the hair of children with parents using cocaine who are exposed to a contaminated environment, with considerable clinical findings (neurological and respiratory abnormalities), fatal poisonings by active ingestion (by hand-to-mouth activity) of powder residues [20,21] or passive crack smoke inhalation [22-24].

In this case report the appearance of the family and their medical records, in addition to the clinical features presented by the child, suggested the possibility of an unhealthy environment and consequently intoxication with drugs of abuse. Her exposure to cocaine in the days preceding her admission was confirmed by an initial positive urinary immunoassay, in addition to symptoms suggesting recent exposure to crack cocaine smoke.

Under the circumstances, analysis of the child's and parent's hair was performed in order to assess previous and chronic exposure to cocaine and other illicit drugs (the clinical consequences of a polyexposure can be very serious). The BZG concentrations found in the proximal and distal extremities of our patient's hair demonstrated repeated exposure to environmental cocaine at least during the last three months. Therefore, the need to be admitted at the pediatric emergency, the medical background of the child and the presence of BZG in her hair can be all explained by a systematic passive exposure to crack cocaine smoke. The BZG concentration in her hair was proportionally higher than the BZG concentration in her parent's hair, who actively smoked crack cocaine. Considering this child's age, the results suggest that passive environmental exposure in young children may have highly toxic, even potential lethal, effects, probably due to their immature metabolizing capacity. In fact, previous cases of symptomatic chronic intoxication by cocaine have been described, but with lower concentration of cocaine in hair in comparison with adult hair concentrations [25-29].

Based on the data from this case and other poisoning cases published by our group and other groups (Table 2) [25-29], we conclude that parental use of cocaine can be reflected in a non-negligible exposure of their progeny, and children show higher concentrations of cocaine in hair and they experience more serious deleterious acute effects, perhaps due to different and slower cocaine metabolism.

**Table 2:** Cases reports of acute intoxication in children exposed passively to cocaine and confirmed by hair analysis.

	Age	Chronic exposure	Benzoylecgonine in hair determined by gas chromatography/mass spectrometry (GC/MS) (ng/mg)
[25]	11 months	Cocaine	Infant's hair
			1 cm to 4 cm: 0.4
			4 cm to 8 cm: 10.5
			Mother's hair
			1 cm to 10 cm: negative for any illicit substance (bleached hair)

[26]	15 months	Cocaine	Infant's hair
			1 cm to 2 cm: 2.2
			2 cm to 7 cm: 7.6
			Sister's hair
			1 cm to 2 cm: negative
			2 cm to 7 cm: 0.5
			7 cm to 20 cm: 1.2
			Mother's hair
			1 cm to 2 cm: 8.2
			2 cm to 7 cm: 5.3
			7 cm to 20 cm: 10.6
			Father's hair
			1 cm to 2 cm: 3.0

[27]	24 months		Infant's hair
			0 cm to 1 cm: 1.9
			1 cm to 15 cm: 17.2^a^

[28]	13 months	Cocaine	Infant's hair
			0.6

[29]	One month	Cocaine	Infant's hair
			2.2
			Mother's hair
			3.4
			Father's hair
			1.7

^a^Mean concentration.

## Conclusion

We conclude that passive exposure to smoked cocaine must be strictly avoided in infants and children. In addition, drug testing in conventional (urine and blood) and non-conventional (hair) matrices should be used routinely in all the children who present with specific or suspicious symptoms of acute and/or chronic exposure to illicit drugs, particularly with suspected drug-using parents, frequently polyusers. Once again, hair analysis proved to be a useful tool in disclosing chronic exposure to drugs of abuse. Furthermore, hair analysis offers the opportunity to periodically examine drug exposure in order to protect these children from significant health and social risks. We advocate hair analysis in all cases of newborns, toddlers and children from risky environments.

## Consent

Written informed consent was obtained from our patient's parents (her legal guardians) for publication of this case report and any accompanying images. A copy of the written consent is available for review by the Editor-in-Chief of this journal.

## Competing interests

The authors declare that they have no competing interests.

## Authors' contributions

EP was the main contributor in writing the manuscript. XJ was an important laboratory technician in biomarkers analyses, and contributed in writing the manuscript. MV analyzed the mother-infant data, reviewed the literature and the final manuscript, and contributed in writing the manuscript. EC was the main laboratory technician in biomarkers analyses. PM analyzed the mother-infant data, and was a contributor in writing the manuscript. MB analyzed the mother-infant data, and was the statistical expert. OV was the pediatrician responsible for coordination of data, and contributed in writing the manuscript. OGA analyzed the mother-infant data, reviewed the literature, and was a major contributor in writing the manuscript. All authors have read and approved the final manuscript.
